# The Autophagic Route of E-Cadherin and Cell Adhesion Molecules in Cancer Progression

**DOI:** 10.3390/cancers13246328

**Published:** 2021-12-16

**Authors:** Manuela Santarosa, Roberta Maestro

**Affiliations:** Unit of Oncogenetics and Functional Oncogenomics, CRO Aviano, National Cancer Institute, IRCCS, 33081 Aviano, Italy

**Keywords:** cancer, carcinoma, E-cadherin, autophagy, adherens junctions, cell adhesion, epithelial to mesenchymal transition, metastasis

## Abstract

**Simple Summary:**

A hallmark of carcinoma progression is the loss of epithelial integrity. In this context, the deregulation of adhesion molecules, such as E-cadherin, affects epithelial structures and associates with epithelial to mesenchymal transition (EMT). This, in turn, fosters cancer progression. Autophagy endows cancer cells with the ability to overcome intracellular and environmental stress stimuli, such as anoikis, nutrient deprivation, hypoxia, and drugs. Furthermore, it plays an important role in the degradation of cell adhesion proteins and in EMT. This review focuses on the interplay between the turnover of adhesion molecules, primarily E-cadherin, and autophagy in cancer progression.

**Abstract:**

Cell-to-cell adhesion is a key element in epithelial tissue integrity and homeostasis during embryogenesis, response to damage, and differentiation. Loss of cell adhesion and gain of mesenchymal features, a phenomenon known as epithelial to mesenchymal transition (EMT), are essential steps in cancer progression. Interestingly, downregulation or degradation by endocytosis of epithelial adhesion molecules (e.g., E-cadherin) associates with EMT and promotes cell migration. Autophagy is a physiological intracellular degradation and recycling process. In cancer, it is thought to exert a tumor suppressive role in the early phases of cell transformation but, once cells have gained a fully transformed phenotype, autophagy may fuel malignant progression by promoting EMT and conferring drug resistance. In this review, we discuss the crosstalk between autophagy, EMT, and turnover of epithelial cell adhesion molecules, with particular attention to E-cadherin.

## 1. Introduction

In normal differentiated epithelial tissue, cell-to-cell contacts are crucial in maintaining tissue integrity and in preventing inappropriate cell proliferation [1,2,3]. Contact-mediated control over cell division is turned off during embryonic development, tissue regeneration and wound healing, and is deregulated in solid tumors [4,5]. Namely, during tumor progression, cell-to-cell junctions are disrupted and malignant epithelial cells switch towards a plastic stem-like phenotype and take on mesenchymal characteristics. This occurs through the reactivation of a phenomenon named epithelial to mesenchymal transition (EMT), a developmental program that takes place during gastrulation to form the mesoderm [5,6,7]. Indeed, EMT features have been demonstrated in several carcinomas, including breast, ovarian, colon, pancreatic, and lung cancers [5,8,9,10,11,12,13], and are associated with tumor aggressiveness and resistance to conventional and target therapies [14,15,16,17,18,19,20,21].

EMT has long been regarded as a rate-limiting mechanism for invasion and metastasis whereby cells are empowered with increased migratory capability as a result of the aberrant activation of mesenchymal transcription factors (EMT-TFs), such as the SNAI, ZEB, and TWIST family members [5,22]. Conversely, recent studies have clarified that the gain of a fully mesenchymal phenotype is not an essential requirement for metastasis [19,23]. It has been shown that the reactivation of the EMT program may lead to an array of phenotypic nuances, from partial to complete loss of cell-to-cell junctions, apicobasal polarity, cytoskeletal remodeling, and induction of mesenchymal markers [6,24]. Indeed, single-cell transcriptome analyses of metastatic clones revealed a predominance of a partial EMT state (pEMT), characterized by the concomitant expression of both epithelial and mesenchymal markers (e.g., E-cadherin and N-cadherin) [25,26,27,28]. On the other hand, the disruption of cell-to-cell contacts may also trigger EMT [5,7,29,30]. In particular, the internalization of cell-to-cell junction proteins has been described to associate with pEMT and to induce collective- and single-cell migration [31].

Autophagy is a physiological, evolutionarily conserved, lysosomal self-degradative process that responds to different stimuli, such as food deprivation, hyperthermia, hypoxia, and xenobiotics [32]. Activation of autophagy leads to the clearance of various cellular components, including damaged organelles, unfolded proteins, and abnormal protein aggregates. The way these cellular components are delivered to the lysosomes defines the different types of autophagy: macroautophagy (hereafter referred to as autophagy), microautophagy, and chaperone-mediated autophagy. Moreover, autophagy is referred to as metabolic autophagy when it is induced to generate nutrients and support general cellular growth, and quality control autophagy if it is activated for cleaning cells from damaged molecules/organelles [33]. Finally, lysosome-independent autophagy has been recently described as secretory autophagy [34].

Autophagy is implicated in numerous pathological conditions including cancer, where it plays a dual role: prevention of tumor development in the early phases of cell transformation; and promotion of cell survival and tumor progression once transformation has occurred [35,36,37]. Autophagy is activated in several tumors [38,39,40,41] and it is frequently described as a mechanism of resistance to various cancer treatments such as chemotherapy, targeted therapy, or immunotherapy [42,43,44,45,46].

The fact that both EMT and autophagy may endow carcinoma cells with metastatic potential and drug resistance suggests a crosstalk between the two pathways. Also, the data indicating that pEMT may be driven by internalization/degradation of cell-to-cell junction proteins supports this link. In this review, we discuss the evidence that supports an interplay between autophagy, EMT, and turnover of cell adhesion molecules in cancer progression, with particular attention to the epithelial cadherin (E-cadherin).

## 2. Methods

On June 2021 a literature search was done by querying the Title/Abstract field of the PubMed database with the following string: (mesenchymal transition OR E-cadherin OR adherens junctions OR tight junctions) AND autophagy AND (cancer OR carcinoma). The search retrieved 454 papers written in English. Only reviews and papers specifically dealing with EMT, E-cadherin, or autophagy in cancers and references therein were selected. The numerous papers in which the expression of E-cadherin, EMT or autophagy markers (irrespectively of the actual activation of autophagy) were reported as mere readouts of drug resistance/response were dropped.

## 3. Cell-to-Cell Contacts, Adherens Junctions and EMT

**Figure 1:**
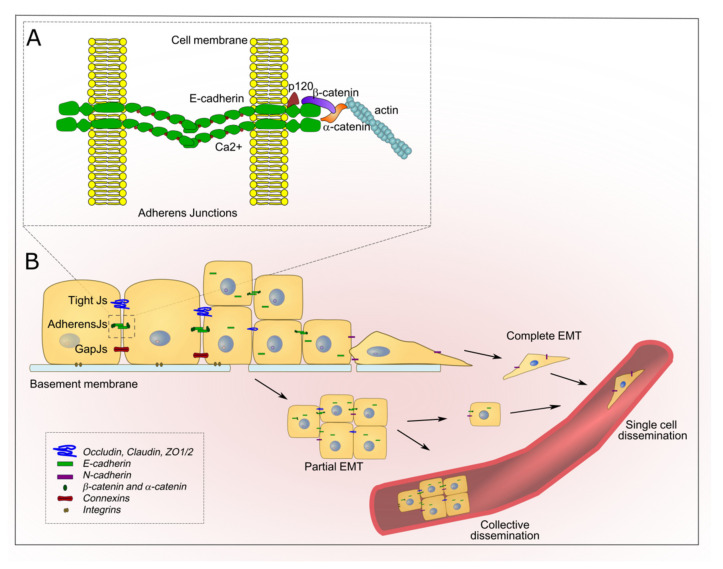
Epithelial tissue organization is dismantled in cancer progression. (**A**) Schematic representation of the transmembrane protein E-cadherin and catenins at the adherens junctions (AJs). The extracellular region of E-cadherin is composed of five Ca^2+^ binding domains and participates in cis and trans interactions. The intracellular juxtamembrane domain (JMD) and the carboxy-terminal domain of E-cadherin interacts with p120- and β-catenin, respectively. β-catenin, in turn, binds to the actin cytoskeleton via α-catenin. (**B**) Changes in the cell-to-cell tight, adherens, and gap Junctions (Js) occur during the transition from the normal epithelial state (left) to partial (pEMT) or complete EMT(right). Tumor cells with EMT phenotypes migrate primarily as single cells, whereas pEMT cells may disseminate by both single and collective pathways.

Cell-to-cell adhesion and apical-basal polarity are essential components of the epithelial phenotype and are maintained by specific adhesion structures, such as adherens junctions (AJs), tight junctions, and desmosomes (Figure 1A).

In polarized epithelial sheets, AJs form a continuous adhesive belt at the apical-lateral interfaces of cell-to-cell contacts [47] and are involved in mechanical tension and tissue integrity [48]. Cadherins, key components of AJs, constitute a large family of cell surface adhesion receptors whose name reflects their calcium-dependent adhesion functions. In fact, classical cadherins are characterized by the extracellular cadherin domains, which are involved in Calcium-mediated cis- and trans-homophilic interactions between cells; a single transmembrane segment; and a cytoplasmic region that interacts with catenin proteins [47,49]. Specifically, the intracellular region of E-cadherin interacts with p120-catenin by the juxtamembrane domain (JMD) and with β-catenin through the C-terminus. β-catenin, in turn, is dynamically linked to the actin cytoskeleton via α-catenin [50,51].

Different members of the cadherin family are expressed in a tissue-specific manner and give rise to strong homophilic interactions that determine the strength of AJs. Heterophilic interactions can also be established, albeit at weaker affinity. For example, E-cadherin can interact with N-cadherin [52,53], which may be neo-expressed in transformed epithelial cells as a consequence of EMT-induced cadherin switch [25,26].

In normal epithelia, interactions among AJs and between AJs and the actin cytoskeleton are dynamic. They are continuously formed, broken, and rearranged to maintain tissue homeostasis and to allow tissue remodeling [54]. This dynamic state is ensured by continuous trafficking of E-cadherin to and from the cell surface-mediated by exocytotic and endocytic pathways [55]. Moreover, downregulation of E-cadherin, together with increased cell-matrix interactions, is essential for budding epithelial morphogenesis. In this process, cells expressing low levels of E-cadherin engage with the basement membrane rather than with other cells, thus allowing epithelia to protrude and develop [56]. AJs remodeling occurs also in pathogenic processes, such as cancer dissemination. In fact, functional inactivation or downregulation of E-cadherin are critical steps in the disruption of epithelial organization and in the metastatic process of carcinomas [5,57].

Several mechanisms may contribute to E-cadherin downregulation, including transcriptional repression and increased degradation [22,30,47,58,59]. In full EMT, E-cadherin is typically transcriptionally repressed by EMT-TFs which, at the same time, induces the expression of mesenchymal genes, including N-cadherin and intermediate filaments [5]. The transcriptional repression of E-cadherin produces single cells capable of invading tumor surrounding tissues and disseminating through circulation (Figure 1B). Metastasis can also occur through collective cell migration whereby small clumps of cells migrate in a coordinated manner in response to environmental stimuli [60]. The high level of intercellular organization required for this type of migration is orchestrated by the cytoskeleton-adherens/tight junctions to produce an anterior-posterior polarity in which a group of cells leads the invasive front [61]. Collective migratory patterns have been described in several carcinomas [31,60,62,63].

Tumors featuring complete EMT tend to disseminate as single cells. Conversely, a pEMT phenotype associates with both collective and single cell migration patterns [31]. For instance, in the circulation of patients with metastatic Non-Small Cell Lung Cancer, single as well as clusters of tumor cells featuring pEMT are detected, indicating that incomplete EMT is key to metastasis in these patients [64]. Similarly, the single-cell transcriptome of primary head and neck tumors demonstrated that cells localized at the edge of the tumor may feature pEMT. Tumors with these phenomena were of higher grade and were more prone to nodal metastasis. Interestingly, this report indicated that pEMT cells fail to express the classical EMT-TFs [65]. An EMT-TF-independent pEMT was also described in pancreatic cancer, where downregulation of E-cadherin was caused by protein internalization rather than transcriptional repression [31]. A moderate expression of E-cadherin, typical of a pEMT, was also required for collective migration and effective metastatization of tumor cells in the murine breast cancer model [66]. The authors show that tumor cells in which the expression of E-cadherin was completely abrogated, although mesenchymal and highly motile, were far less efficient in generating metastasis than cells that retained residual levels of E-cadherin [66,67].

Collectively, these data indicate that the disaggregation of AJs and the gain of a hybrid epithelial-mesenchymal phenotype, typical of pEMT, both foster tumor progression. Whether the simultaneous expression of epithelial and mesenchymal cadherins is due to an interaction between these molecules and a consequent signaling reprogramming remains to be elucidated [25,26].

### Functions and Regulation of E-Cadherin

E-cadherin, encoded by the Cdh1 gene, is essential for normal embryonic development and epithelial functions, as shown by the early lethality of Cdh1 knockout mice [68]. Inactivation of E-cadherin in adult epithelia has a tumor promoting effect. In fact, simultaneous inactivation of Cdh1 and p53 in the breast epithelium of a conditional mouse model produced an invasive lobular carcinoma [69]. In humans, germline mutations of the CDH1 gene predispose to lobular breast and diffuse gastric cancers [70,71]. Somatic CDH1 mutations in these tumors are also common [72,73,74,75]. CDH1-associated lobular breast and diffuse gastric cancers are characterized by small, non-cohesive epithelial cells individually dispersed or organized in a single-file linear pattern in a fibrous stroma [76,77]. Although most data support the notion that CDH1 is a tumor suppressor, Kleer and coworkers have shown that in inflammatory breast cancers, E-cadherin seems to exert an opposite role [78]. In these aggressive tumors, E-cadherin was highly expressed in both primary lesions and intralymphatic tumor emboli, thus challenging the common notion that E-cadherin loss correlates with poor prognosis in carcinomas.

The expression of E-cadherin is finely controlled at both transcriptional and post-transcriptional levels. E-cadherin transcriptional regulation and biogenesis have been extensively reviewed elsewhere (see for example [54,79,80,81,82]). This review focuses primarily on the mechanisms of regulation of E-cadherin turnover.

The availability of E-cadherin at cellular junction sites is determined by the rates of new protein synthesis, protein internalization, degradation, and recycling. The half-life of E-cadherin has been estimated in the range of a few minutes to hours [83,84,85]. The rapid changes in cell adhesion required during cellular movements and tissue remodeling are sustained by cyclical endocytosis and exocytosis of cadherin molecules [47]. In fact, E-cadherin undergoes constant turnover due to trafficking between the cell surface and the cytoplasm, where it can be stored, rapidly redirected to the membrane, or degraded [54,58,83,86]. In particular, it has been demonstrated that the fraction of E-cadherin not engaged in intra- or inter-cellular bonds is predominantly endocytosed through a pathway resembling macropinocytotic [87]. Finally, pioneering experiments have shown that, upon extracellular calcium depletion, E-cadherin undergoes rapid endocytic internalization and AJs were dismantled but readily reformed after the restoration of normal Ca^2+^ levels [58,88]. Thus, E-cadherin trafficking controls both the stability and plasticity of AJs.

The trafficking of E-cadherin is significantly affected by several post-translation modifications such as phosphorylation, ubiquitination, and glycosylation, as well as protein interactions [89]. For example, the binding between p120 and E-cadherin masks the endocytic signal located in the proximity of the E-cadherin JMD domain, thus preventing endocytosis and lysosomal degradation and maintaining E-cadherin at the plasma membrane [89,90,91,92]. The JMD of E-cadherin also contains sites for tyrosine phosphorylation-mediated ubiquitination by the E3 ubiquitin ligase Hakai (encoded by the CBLL1 gene), which targets E-cadherin for endocytosis and proteasomal or lysosomal degradation [93,94]. Finally, the binding of E-cadherin to p120 has been found to be crucial in the constitutive dimerization of E-cadherin, which is instrumental for the formation of AJs [95]. Interestingly, the degradation of E-cadherin promoted by phosphorylation of the JMD domain is thought to be an early event that precedes transcriptional inhibition of CDH1 during EMT [96].

## 4. Autophagy and EMT

The autophagic process is finely orchestrated by a set of more than 40 autophagy-associated proteins (ATGs), all of which are highly conserved in eukaryotes [97] and have been described in detail in several reviews (see for example [98,99,100]). The core machinery of autophagy, represented by ATG1/ULK, ATG9, ATG14, ATG2, ATG18/WIPI, ATG12, ATG5, ATG16L1, and ATG8/LC3, participates in the formation of the lipid-bilayer membrane sack that traps the cytoplasmic cargo by stretching and bending to form the autophagosome that eventually fuses with lysosomes for degradation [32]. The process requires the recruitment to the outer surface of the membrane of the ATG12-ATG5-ATG16L1 complex, which is formed by the sequential action of the ATG4 protease and the E1-like and E2-like enzymes ATG7 and ATG3, respectively. This complex then contributes to the conjugation of LC3-I with the membrane lipid phosphatidylethanolamine (PE) to generate LC3-II. LC3-II in turn associates with the membrane of autophagosomes and interacts with the LC3-interacting region (LIR) of specific adaptor proteins such as SQSTM1/p62, NBR1, NDP52 [101]. LC3-II is considered a hallmark of autophagy activation, and the ratio between LC3-II and the non-lipidated form of LC3-I protein is considered a readout of autophagic flux [102].

During metastatic progression, autophagy endows cancer cells with the ability to overcome intracellular and environmental stress stimuli such as anoikis, nutrient deprivation, hypoxia, and cytotoxic drugs [38,103] and to enhance cell migration [104,105,106,107]. These same stimuli can promote EMT and, indeed, in some experimental models, EMT cells rely on autophagy for metastatic dissemination [108]. For example, subpopulations of a lung cancer cell line that acquired the T790M EGFR resistance mutation have been described to exhibit an EMT phenotype and to require autophagy for survival [109].

For these reasons, the connection of autophagy with EMT has long been investigated, although it is still a matter of debate.

Several studies suggest that the activation of autophagy promotes disaggregation of the epithelium and, consequently, mesenchymal transition and migration. On the contrary, other studies indicate that an autophagic-mediated degradation of EMT-TFs induces the reversion of EMT [110].

A case in point is the role of autophagy in modulating the induction of EMT by TGF-β: autophagy has been reported to assist the TGF-β/SMAD signaling in the induction of EMT in cancers and normal tissues [111,112,113,114,115,116,117]. Namely, autophagy promotes the overexpression of an E3 ubiquitin ligase, HERC3, that triggers the degradation of the inhibitory Smad SMAD7, thus unleashing the TGF-β/SMAD2/3 pathway, [118]. On the other hand, it has been shown that TGF-β1-induced autophagy has different outcomes in terms of pancreatic cancer progression depending on SMAD4 status [119].

The inhibition of autophagy, chemically or through the knockout/knockdown of autophagy core genes such as Beclin-1 or ATG7, increases the expression of epithelial markers, whereas its induction produces opposite effects [111,120,121,122,123]. In particular, Chen and colleagues showed that autophagy induced by peritumoral monocytes promotes NFKB-mediated SNAI1 activation and, thereby, EMT [124]. Conversely, other works reported a role for autophagy in fostering epithelial features, for example, by inducing E-cadherin localization to the plasma membrane, by limiting the expression of EMT-TFs [125,126,127,128]. Moreover, autophagy is linked through a negative feedback loop to the Wnt/β-catenin pathway, a known inducer of EMT. Specifically, β-catenin limits autophagy activation in physiological nutrient-rich conditions [129]. On the other hand, in colorectal cancer, autophagy drives the degradation of β-catenin and Dishevelled2 (DVL2) proteins, thus hampering Wnt/β-catenin induced EMT [129,130,131,132]. Conversely, the inhibition of the autophagy-mediated degradation of DVL2 results in EMT and augmented aggressiveness of hepatocellular carcinoma [133].

The autophagy-mediated control of EMT-TFs occurs in part through the autophagy adaptors, which organize the delivery of specific proteins to autophagosomes and are themselves the target of autophagy-mediated degradation. In glioblastoma, active autophagy promotes the degradation of SNAI1 via binding to the autophagy adaptor SQSTM1/p62 [134]. In mouse embryonic fibroblasts and human cancer cell lines, autophagy deficiency leads to the accumulation of the SQSTM1/p62 which by binding TWIST1 and SNAI1, leads to their stabilization and thereby increased migration and loss of epithelial markers [135,136,137,138]. Likewise, accumulation of SQSTM1/p62 activates the NFκB/RELA pathway, which in turn activates the transcription of ZEB1 and SNAI1 in RAS-mutated cancer cells and fibrotic interstitial lung cells, respectively [127,139]. Moreover, SQSTM1/p62 has been described to cooperate with NRF2 signaling to sustain the expression of EMT-TFs in a mesenchymal glioblastoma subtype [140]. It is noteworthy that overexpression of SQSTM1/p62 is a common finding in several carcinomas where it correlates with EMT and metastasis and it is considered a negative prognostic factor [135,141,142,143].

The above studies suggest a complex interplay between autophagy and EMT, the outcome of which appears to depend on the cellular and environmental context, such as cell type, oncogene dependency, and type of stimuli that induce autophagy, etc.

### 4.1. Autophagy Affects the Turnover of Proteins Involved in Cell-to-Cell and Cell-to-Matrix Interactions

A large body of evidence points to a link between autophagy, adhesion molecules and cell motility. Although activation of autophagy may hamper cell motility in some instances, as in the case of the autophagy-mediated degradation of β1 integrins that results in impaired migratory capability of HeLa cells [105], there is ample evidence that autophagy spurs metastasis.

**Figure 2:**
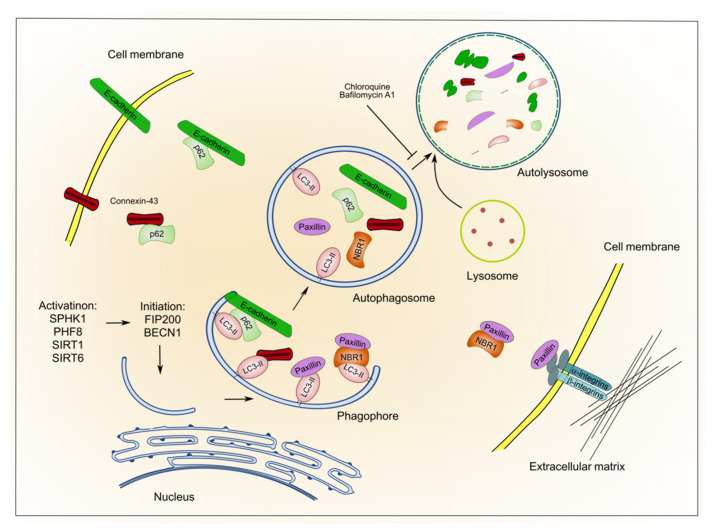
Autophagy-mediated degradation of cell adhesion molecules. The scheme reports only the molecules described in the text.

As mentioned above, the disruption of AJs as a consequence of E-cadherin downregulation can trigger EMT and the gaining of disseminative properties [31,96]. For instance, the downregulation of E-cadherin can trigger a reversible pEMT through the induction of snail family members [144]. Mounting evidence suggests that autophagy modulates the degradation of proteins involved in cell-to-cell and cell-to-matrix interactions (Figure 2).

The control of E-cadherin expression at the plasma membrane and in the cell-to-cell adhesion loci takes place thanks to E-cadherin internalization and recycling or degradation. Several reports demonstrate that E-cadherin is downregulated through autophagy. For example, SPHK1, a regulator of sphingolipid metabolism, by binding TRAF2 (TNF receptor-associated factor 2) and Beclin-1, activates autophagy and thereby induces the degradation of E-cadherin, EMT, and the metastatic progression of hepatocellular carcinoma [145]. Zhou and colleagues have also shown that FIP200 (encoded by the RB1CC1 gene), a key player in autophagosome formation, following induction by the histone demethylase PHF8 (a.k.a KDM7B or JHDM1F), activates autophagy and autophagy-mediated degradation of E-cadherin [146].

Similarly, the histone deacetylases SIRT1 and SIRT6 promote the metastatic potential of melanoma and hepatocellular carcinoma cells, respectively, by deacetylating Beclin-1 and accelerating autophagic degradation of E-cadherin [123,147]. We have recently demonstrated that the inhibition of autophagy restores E-cadherin expression and suppresses cell migration and invasion. Moreover, our results indicate that E-cadherin is delivered to autophagosomes for degradation by SQSTM1/p62 upon autophagy activation. This results in the disruption of the AJs between breast cancer cells, thus establishing a link between the control of E-cadherin turnover and the stability of AJs and, hence, the epithelial integrity [41].

The role of autophagy in the dynamics of focal adhesions (FA) during cell migration and invasion has also been demonstrated. It has been shown that autophagy activation is required for the disassembly of FA. Namely, paxillin (encoded by the PXN gene), a key component of FA, is degraded following interaction, direct or NBR1-mediated, with LC3 [148,149]. Also connexin-43 (GJA1 gene), a gap junction protein, once internalized is degraded by autophagy after SQSTM1/p62 binding and delivery to autophagosome [150] and, interestingly, connexin-43 has been observed to be downregulated in breast and prostate cancers [151,152]. Finally, the restoration of gap junctions between tumor cells has been reported to restrain the EMT process and metastasis [153]. However, we ought to mention that, in other contexts, the gap junctions between tumor cells and stroma have been reported to promote invasion [154,155].

### 4.2. Perturbation of Cell Adhesion by Secretory Autophagy

Besides acting as an inner mechanism, the autophagy-mediated secretion, by impinging on the tumor microenvironment, may affect tumor cells adhesion and invasion.

Autophagy is not only involved in the degradation of intracellular components, but also mediates unconventional forms of secretion in what is called “secretory autophagy” [34,156,157]. In this process, instead of fusing with lysosomes, autophagosomes fuse with the plasma membrane and release their cargo contents into the extracellular environment. Secretory autophagy contributes to the secretion of molecules that fail to enter the conventional secretory system due to the lack of the leader signal sequence that directs proteins to the endoplasmic reticulum-Golgi route of secretion [156]. Even cytoplasmic organelles, viruses, and bacteria can be secreted by the autophagic machinery [158,159,160,161].

The mechanisms that govern autophagy-mediated degradation or autophagy-dependent secretion are still poorly defined, although some evidence suggests that the two autophagic routes rely on different receptors [162]. Clearly, the activation of secretory autophagy in tumor and stromal cells results in the release in the extracellular space of a number of factors, including inflammatory cytokines and chemokines (e.g., interleukin 1β, CXCL8, LIF), thus impacting on the tumor microenvironment [163,164,165]. Interestingly, depletion of autophagy-related genes in HRAS-driven epithelial tumor cells diminishes the secretion of pro-invasive molecules (Interleukin-6, metalloproteinases 2, WNT-5A), inhibits cell motility, and reduces metastatic potential [106].

The secretion of matrix components also seems to rely, at least in part, on autophagy, since ATG7 deficiency results in reduced expression of type 1 collagen, fibronectin 1, and periostin [166]. The same holds true for TGF-β1, whose secretion in human and murine fibroblasts and macrophages are fully abrogated after knockdown of ATG5 [167].

### 4.3. Cell Adhesion Controls Autophagy Activation

Epithelial cells are susceptible to several mechanical stimuli resulting from the cell-to-cell interactions and the extracellular matrix (e.g., composition, stiffness, and density), as well as fluid pressure and lateral stretch and compression required for the physiological function of several organs [168].

Changes in mechanical force can be sensed at cell adhesion junctions where they activate signaling pathways that mediate rearrangements in both the actomyosin cytoskeleton and cell-to-cell contacts [169,170,171,172]. Tensile forces act in the morphogenesis of embryonic tissues as well as in adult tissues, where they are involved in maintaining the barrier of epithelial and endothelial monolayers [172,173,174]. Interestingly, they can also promote safeguard processes such as autophagy [175,176,177,178]. In fact, Bayes and colleagues have demonstrated LKB1 (liver kinase B1)- mediated activation of AMPK (AMP-activated protein kinase), a master regulator of energy homeostasis and autophagy, through the action of tensile forces on E-cadherin, confirming a critical link between cell-adhesion and autophagy [179].

Moreover, autophagy is activated as a protection mechanism to preserve cells from anoikis, a form of programmed cell death typically triggered in anchorage-dependent cells upon detachment from the extracellular matrix [180,181]. Therefore, autophagy affects cell-to-cell adhesion and vice versa the disruption of cell adhesion activates autophagy, adding support to the concept that cell adhesion and autophagy are functionally connected.

## 5. The Prognostic Role of E-Cadherin and Autophagy in Cancer

In the last decades, numerous studies have investigated the prognostic implications of the modulation of the expression of E-cadherin in carcinomas, unfortunately yielding inconclusive results [182,183,184,185,186,187,188]. These apparent discrepancies, observed between studies even with the same tumor histotype, are likely due to differences in E-cadherin staining protocols and scoring criteria [188].

Nonetheless, meta-analyses seem to support a positive association between E-cadherin expression and prognosis at least in gastric, pancreatic, oral, breast, and prostate carcinomas [188,189,190,191,192]. As for autophagy, several studies indicate an augment of the autophagic flux in malignant tumors and metastasis compared to normal tissues. Although it is challenging to determine autophagy flux in tumor specimens, indirect markers of autophagy, such as the punctuate staining for LC3B as a readout of autophagosome assembly, are commonly used. These studies demonstrated an increased autophagic activity in various types of tumors, including breast, gastric and renal carcinomas, and especially in metastatic lesions [38,39,40,193,194]. For instance, in breast cancer, an increase in LC3B punctuate staining was observed in lymph node metastasis and was associated with metastatic potential and reduced survival [38,195].

Finally, a microarray study indicated that the aggressive behavior of glioblastoma multiforme was associated with the enrichment of an autophagy gene signature [104,196].

## 6. Conclusions and Future Directions

The mechanisms that control epithelial homeostasis play a fundamental role in maintaining epithelial cell functions and preventing transformation. In this context, cell-cell junctions, which are required for both cell adhesion and tissue dynamics, are of fundamental importance. Several processes can compromise epithelial integrity, thereby promoting tumor development and progression, including EMT (complete or partial) and autophagy. How these interconnected networks influence carcinoma malignant evolution is still unclear, but appears to depend at least in part on the type of stimulus, cell histotype, and cancer stage.

Autophagy is known to play a paradoxical role in normal and in tumor cells. One theme that emerges in this review is the contribution of degradation of adhesion molecules, particularly E-cadherin, to the pro-metastatic functions exerted by autophagy in transformed cells. Which are the molecular switches that convert autophagy from an anti-tumor pathway to an ally of cancer progression remains unknown. It is also unclear how autophagy-mediated turnover of adhesion molecules affects the transcription of genes involved in EMT.

There is growing preclinical and clinical evidence that inhibition of autophagy has therapeutic relevance, particularly in potentiating the efficacy of cancer therapies [197,198]. A deeper understanding of the interplay between cell adhesion, EMT, and autophagy could reveal important tumor vulnerabilities and help in optimizing anti-autophagy approaches in the clinical setting.
